# Development of Ag-ZnO/AgO Nanocomposites Effectives for *Leishmania braziliensis* Treatment

**DOI:** 10.3390/pharmaceutics14122642

**Published:** 2022-11-29

**Authors:** Rafaela Miranda Barbosa, Malu Mateus Santos Obata, José Rodrigues do Carmo Neto, Rhanoica Oliveira Guerra, Anna Victória Bernardes e Borges, Rafael Obata Trevisan, Letícia Cirelli Ruiz, Júlia de Moura Bernardi, Ana Carolina de Morais Oliveira-Scussel, Sarah Cristina Sato Vaz Tanaka, Fernanda Bernadelli de Vito, Fernanda Rodrigues Helmo, Thaís Soares Farnesi de Assunção, Juliana Reis Machado, Carlo José Freire de Oliveira, Virmondes Rodrigues Júnior, Anielle Christine Almeida Silva, Marcos Vinicius da Silva

**Affiliations:** 1 Department of Microbiology, Immunology and Parasitology, Institute of Biological and Natural Sciences, Federal University of Triângulo Mineiro, Uberaba 38025-180, Minas Gerais, Brazil; 2 Department of Bioscience and Technology, Institute of Tropical Pathology and Public Health, Federal University of Goias, Goiania 74690-900, Goiás, Brazil; 3 Department of Medical Clinical, Institute of Health Sciences, Federal University of Triângulo Mineiro, Uberaba 38025-180, Minas Gerais, Brazil; 4 Department of General Pathology, Federal University of Triângulo Mineiro, Uberaba 38025-180, Minas Gerais, Brazil; 5 Laboratory of New Nanostructured and Functional Materials, Physics Institute, Federal University of Alagoas, Maceió 57010-000, Alagoas, Brazil; 6 Postgraduate Program of the Northeast Biotechnology Network (RENORBIO), Federal University of Alagoas, Maceio 57010-000, Alagoas, Brazil

**Keywords:** leishmaniasis, nanocomposites, therapeutics, zinc oxide, silver oxide, silver doped, *Leishmania braziliensis*

## Abstract

Tegumentary leishmaniasis (TL) is caused by parasites of the genus Leishmania. *Leishmania braziliensis* (*L.b*) is one of the most clinically relevant pathogens that affects the skin and mucosa, causing single or multiple disfiguring and life-threatening injuries. Even so, the few treatment options for patients have significant toxicity, high dropout rates, high cost, and the emergence of resistant strains, which implies the need for studies to promote new and better treatments to combat the disease. Zinc oxide nanocrystals are microbicidal and immunomodulatory agents. Here, we develop new Ag-ZnO/xAgO nanocomposites (NCPs) with three different percentages of silver oxide (AgO) nanocrystals (x = 49%, 65%, and 68%) that could act as an option for tegumentary leishmaniasis treatment. Our findings showed that 65% and 68% of AgO inhibit the extra and intracellular replication of *L.b.* and present a high selectivity index. Ag-ZnO/65%AgO NCPs modulate activation, expression of surface receptors, and cytokine production by human peripheral blood mononuclear cells toward a proinflammatory phenotype. These results point to new Ag-ZnO/AgO nanocomposites as a promising option for *L. braziliensis* treatment.

## 1. Introduction

Leishmaniases are a set of neglected diseases caused by intracellular protozoa of the genus *Leishmania* spp., transmitted by female sandflies during blood feeding and are endemic in underdeveloped tropical and subtropical regions of the world, denoting an important public health problem [1,2]. Twenty-one species of *Leishmania* are known to have the ability to infect humans and cause different clinical manifestations depending on the species and virulence of the parasite as well as the immune response of the host [3,4]. Leishmaniasis is divided into visceral leishmaniasis, a more severe form that affects the individual’s viscera, and cutaneous leishmaniasis (TL), which can affect the skin and/or mucosa of infected individuals [1,5]. TL is characterized by single or multiple, difficult-to-heal lesions that mainly affect the skin of exposed areas of the body, while in mucocutaneous leishmaniasis (MCL), disfiguring and potentially fatal lesions are observed on the skin and oral and nasal mucosa [4,6].

According to the World Health Organization (WHO), it is estimated that the number of new annual cases of TL varies from 0.7 to 1.2 million [7], and *Leishmania brasiliensis* (*L.b*) is an important species that can cause LT and MCL [8,9,10]. In addition to the physical damage caused by TL, there are also problems in the social relationships of those affected, as there is the stigma related to injuries and scars that bring the false conception of being a contagious disease that can be transmitted by physical contact [10,11].

Furthermore, the problems related to LT extend to treatments. First, for adequate treatment, it is necessary that the affected receive an individualized evaluation to establish clinical, genetic and immune aspects of the host, as well as the characteristics of the parasite [12,13]. Some cases of LT do not require treatment, and the lesions heal spontaneously; however, when this does not occur, drug therapies such as amphotericin B deoxycholate, lipid formulations of amphotericin B, pentamidine, pentavalent antimony and miltefosine are the most used to treat patients [12,14]. Additionally, due to the difficulty in choosing the treatment, the high cost, toxicity and side effects result in significant rates of treatment abandonment, causing the lesions to persist and progress in patients, in addition to contributing to the emergence of parasites resistant to the available drugs in the market [15,16]. This added to the fact that TL is endemic mainly in underdeveloped tropical regions where health services are insufficient to adequately serve the entire population the TL problem becomes even worse [2].

The use of nanoparticles (NPs) has proven to be an important ally to solve these challenges. Mainly metallic and metallic oxide NPs, such as zinc oxide (ZnO), silver (Ag^2+^), silver oxide (AgO) and Ag-doped ZnO (Ag/ZnO), have important biological properties, such as antimicrobial [17,18,19], antitumor [20,21,22], immunomodulatory properties [23,24], and many others properties such as anti-inflammatory, antidiabetic, antioxidant, antiviral orthopedic implants, bone healing, wound healing and cardioprotective activity [25,26]. Furthermore metallic nanoparticles/nanocomposites present antiparasitic activity against *Trypanosoma cruzi* [27,28], *Toxoplasma* spp. [29,30] and leishmanicidal activity [31,32,33,34]. Along with this, it is known that NPs from the same material, but with differe+nt shapes and sizes, can present different biological properties [35,36], and that a set of NPs can form nanocomposites (NCPs) that can be promising in the treatment of diseases caused by pathogens [37].

With that in mind, the present work aimed to study ZnO nanocrystals (NCs) and NCPs of Ag-ZnO NCs plus x AgO NCs (x = 49%, 65% and 68% being named as ZnO:5Ag, ZnO:9Ag and ZnO:11Ag, respectively) to evaluate the possible leishmanicidal effect on the promastigote forms of *L.b*. Furthermore, it was correlated with immune modulation in peripheral blood mononuclear cells (PBMCs) from healthy donors. Our findings showed that the target NCPs of the study are promising candidates for new therapeutic options for TL cases, considering that they have leishmanicidal and immunomodulatory activity in our in vitro studies.

## 2. Materials and Methods

### 2.1. Synthesis and Characterization of the Ag-ZnO/AgO Nanocomposites

The nanocomposites Ag-ZnO/AgO were synthesized by a method which is under a patent application (BR 10 2018 0077147). The samples used in this work were identified with the names ZnO:5Ag, ZnO:9Ag and ZnO:11Ag. The physical properties of the nanocomposites were investigated by X-ray Diffraction (XRD) and electronic scanning microscopy (SEM). XRD patterns were measured by a Shimadzu XRD-6000 diffractometer with a Cu-target radiation (λ = 0.154 nm). All measurements were performed on powder samples. The SEM images were obtained by scanning electron microscopy (SEM; Carl Zeiss SMT Ltd., EVO MA 15, Oberkochen, Germany).The samples in powders were fixed using carbon tapes on aluminum stubs and covered with gold. All characterizations were performed at room temperature.

### 2.2. RAW 264.7 and Leishmania braziliensis Cultivation

To determine the leishmanicidal activity, strains of *L.b* (IOCL-566) were maintained in Schneider medium [with 10% fetal bovine serum (FBS) and 40 μg/mL gentamicin] at 26°C in a biochemical oxygen demand (BOD) oven. To carry out the experiments, the parasites were used in the stationary phase of growth.

For the toxicity tests, RAW 264.7 murine macrophages maintained in Roswell Park Memorial Institute (RPMI) medium (with 10% FBS and 40 μg/mL gentamicin) at 37 °C and 5% carbon dioxide (CO_2_) were used. Maintenance was performed every two days or when cells reached 80% confluence in the bottle.

### 2.3. Anti-L. braziliensis Activity against Axenic Promastigotes, RAW 264.7 and Intracellular Amastigote

*L.b* (2.5 × 10^6^/mL) was added to 96-well plates followed by treatment with ZnO nanocrystals, ZnO:5Ag, ZnO:9Ag and ZnO:11Ag NCPs at concentrations of 6.25 to 50 µg/mL. The reference drug amphotericin B was used at concentrations of 0.16 to 4 μg/mL as a negative control, and untreated *L.b* was used as a positive control. In parallel, RAW 264.7 cells (2.5 × 10^6^/mL) were placed in 96-well culture plates, and after cell adhesion (4 h), the supernatant was discarded, and the treatments were added.

After 24 h of treatment with *L.b* and macrophages, 5 μL of resazurin (C12H7NO4) at a concentration of 2.5 mg/mL was added to each well, and fluorescence was evaluated using EnSpire^®^ (Perkin Elmer, Rodgau, Germany) at 550–590 nm. Intracellular *L.b*. amastigotes were evaluated by staining with DAPI and IgG anti-*Leishmania*+anti-IgG-FITC and counting at least 200 cells/treatment. RAW 264-7 were infected with *L.b.* (MOI 10:1) for 12 h and treated with different nanoformulations at doses of 50, 25 and 12.5 μg/mL for 72 h.

### 2.4. L. braziliensis Promastigote Growth Curve

*L.b* promastigotes (2.5 × 10^6^/mL) in the stationary phase were added to a 48-well culture plate, followed by the addition of ZnO nanocrystals, ZnO:5Ag NCPs (25 μg/mL), and ZnO:9Ag and ZnO:11Ag NCPs (6.25 to 25 μg/mL). Amphotericin B at 2 μg/mL was used as a negative control, and untreated *L.b*. was used as a positive control. The parasites were counted in a Neubauer chamber after fixation with ice-cold 0.2% paraformaldehyde solution (PFA) every 24 h for 5 days.

### 2.5. Isolation and Treatment of Human Peripheral Blood Mononuclear Cells (PBMCs)

PBMCs from healthy voluntary donors were obtained from peripheral blood using Ficoll-Paque PLUS^®^ (density 1077 g/mL) following the manufacturer’s recommendations. PBMCs were added to RPMI medium (supplemented with 10% FBS and 40 μg/mL gentamicin) to a final concentration of 2 × 10^6^ cells/mL. Then, 250 μL of this solution was added to each well in 48-well plates, followed by treatment with ZnO nanocrystals, ZnO:5Ag, ZnO:9Ag and ZnO:11Ag NCPs at concentrations of 6.25 to 50 μg/mL. Cultures of untreated PBMCs were used as a negative control, and PBMCs stimulated with anti-CD3 and anti-CD28 were used as positive controls. The treated and untreated PBMCs were kept for 72 h at 37 °C with 5% CO_2_, and after that time, the supernatant was collected and stored at −20 °C for cytokine and nitric oxide (NO) analysis.

### 2.6. Immunophenotyping

After the treatment, PBMCs were labeled using four different antibody mixes (all BD Biosciences, San Jose, CA, USA) following the manufacturer’s recommendations: anti-CD4 (PE-Cy7), anti-CD8 (AF-488), anti-CD69 (PE) and anti-annexin-V (APC); anti-CD4 (PE-Cy7), anti-CD8 (AF-488), anti-TNFR1 (PE) and anti-TNFR2 (APC); anti-CD4 (PE-Cy7), anti-CD8 (AF-488), anti-CD210 (PE) and anti-CD73 (APC); anti-CD4 (PE-Cy7), anti-CD8 (AF-488) and anti-PD-1 (PE). A total of 50,000 events were then acquired by a BD FACSCanto^®^ II (BD Biosciences) flow cytometer and analyzed by FlowJo software 10.0.7 (TreeStar Inc., Ashland, OR, USA).

### 2.7. Cytokines and Nitric Oxide (NO) Production

Supernatants from PBMCs were analyzed to evaluate the production of IL-10, TNF-α, IFN-γ (R&D Systems, Minneapolis, MN) and IL-4 (BD Biosciences, Franklin Lakes, NJ, USA) by ELISA. NO production was estimated using modified Griess reagent (Sigma–Aldrich^®^, Saint Louis, MO, USA), both according to the manufacturer’s recommendations. At the end of the experiment, analysis was performed using EnSpire™ Multilabel Reader and InSpire Manager software version 3.0 at 450 nm for cytokine analysis and 540 nm for NO analysis.

### 2.8. Statistical Analysis

The experiments were performed in triplicate, and statistical analyses were performed using GraphPad Prism^®^ version 7.0 software, considering the value of *p* < 0.05 as statistically significant. The selectivity index of each nanocrystal and NCPs was calculated by the ratio between the CC50 of RAW 264.7 and the IC50 of *L.b* (16).

## 3. Results

### 3.1. Structural and Morphological Properties of Ag-ZnO/AgO Nanocomposites

**Figure 1:**
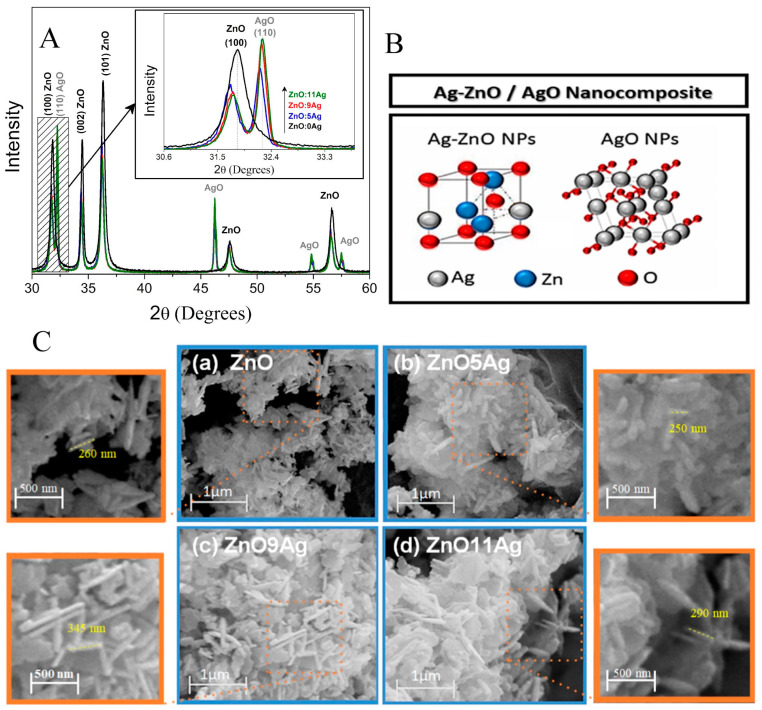
Characterization of Ag-ZnO/AgO Nanocomposites. (**A**) X-ray patterns of ZnO nanocrystals and Ag-ZnO/AgO Nanocomposites (ZnO:5Ag, ZnO:9Ag and ZnO:11Ag). Inset shows the alterations of the ZnO and AgO main peaks due to the Ag concentration. (**B**) Representation of nanocomposite formed by Ag-doped ZnO nanocrystals and AgO nanocrystals. (**C**) Scanning microscopy images of ZnO nanocrystals and Ag-ZnO/AgO Nanocomposites (a) ZnO (b) ZnO:5Ag (c) ZnO:9Ag and (d) ZnO:11Ag and a magnification was performed to visualize the dimensions of the rods.

The structural and morphological properties of the Ag-ZnO/AgO nanocomposites were investigated by XRD patterns and SEM images shown in Figure 1. Figure 1A shows the XRD diffractograms in which it is observed characteristic diffraction patterns of wurtzite ZnO nanocrystals (JCPDS Card No. 36-1451) for all samples and characteristic diffraction of AgO nanocrystals for samples (ZnO:5Ag, ZnO:9Ag and ZnO:11Ag). An amplification was performed to investigate the alterations of the ZnO main peak and the increase in the intensity of the main diffraction peak of AgO with the increase in the Ag concentration, as shown in inset. The shift to smaller angles relative to the ZnO peak (100) with Ag concentration, confirms the substitutional incorporation of silver ions in place of zinc into the ZnO crystalline structure, since Ag^+2^ has an ionic radius (1.26 Å) greater than Zn^+2^ (0.74 Å). It is observed that the peak characteristic diffraction of AgO nanocrystals increases the intensity with Ag concentration. The AgO NCs percentage formed was 49%, 65% and 68% to samples ZnO:5Ag, ZnO:9Ag and ZnO:11Ag, respectively. These results confirmed the formation of nanocomposite of Ag-doped ZnO with AgO nanocrystals.

Figure 1B shows the representation of the constituents of nanocomposite: Ag-doped ZnO nanocrystals and AgO nanocrystals, for each samples has different compositions, 51% of Ag-ZnO and 49% of AgO to sample ZnO:5Ag, 35% of Ag-ZnO and 65% of AgO to sample ZnO:9Ag and 32% of Ag-ZnO and 68% of AgO to sample ZnO:11Ag. Figure 1C shows the SEM images of ZnO nanocrystals (a) and Ag-ZnO/AgO Nanocomposites for samples (b) ZnO:5Ag, (c) ZnO:9Ag and (d) ZnO:11Ag. Observed in the samples containing Ag that when incorporating Ag into ZnO rods there was an increase in length and that the appearance of planar morphology is from AgO nanocrystals, reinforcing the presence of the mixture of nanocrystals forming the nanocomposite. In order to visualize the dimensions of the rods, a magnification was performed.

### 3.2. The NCPs Showed High Selectivity Index for L. braziliensis

Although nanomaterials have different applications in biomedical research, their toxicity in different kinds of cells must be evaluated to ensure their safety [38,39]. Therefore, the resazurin assay was performed to evaluate not only the leishmanicidal activity of nanomaterials in *L.b* strains but also the cytotoxic activity in murine RAW 264.7 ATCC macrophages. Subsequently, with the data from these results, the selectivity index of each nanoformulation was determined.

Thus, it is noted that the nanomaterials tested were more toxic to *L.b* when compared to RAW 264.7 cells (Table 1), inhibiting 50% of promastigotes at doses lower than those necessary to promote 50% inhibition of healthy macrophages. Among the nanoformulations studied, ZnO:9Ag and ZnO:11Ag NCPs showed a selectivity index greater than 10 and, for this reason, showed considerable potential to fight the pathogen [40] when compared to healthy cells used in this study.

### 3.3. ZnO nanocrystals, ZnO:9Ag and ZnO:11Ag NCPs Reduce the Proliferation of L. braziliensis Promastigotes

**Figure 2:**
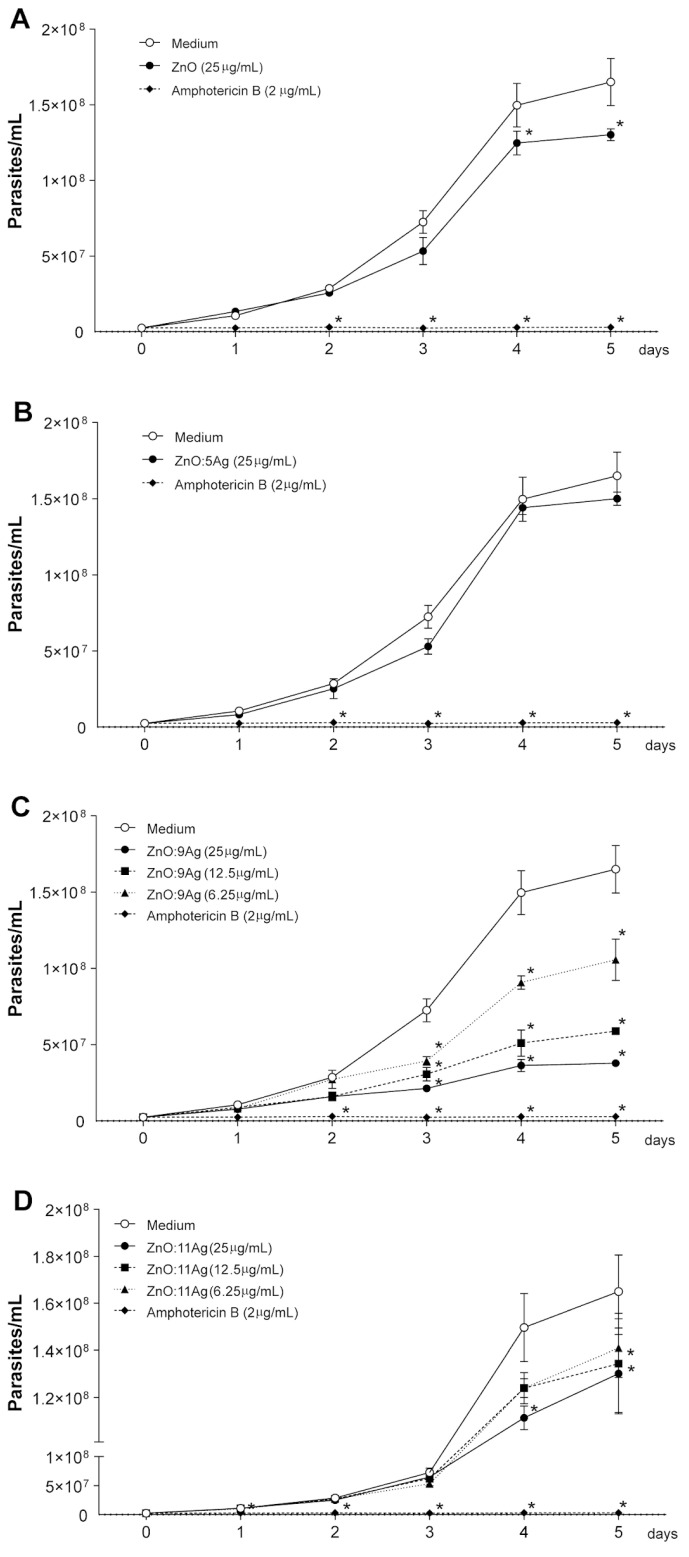
Evaluation of parasitic proliferation when treated with different nanoformulations. The inhibition of proliferation of *L.b* treated with the different nanomaterials was evaluated by counting the parasites in a Neubauer chamber after fixation with ice-cold 0.2% PFA every 24 h for 5 days. The results are expressed as the mean ± SEM (standard error of the mean), and two-way ANOVA of multiple comparisons was applied, with values of *p* < 0.05 (*) being considered significant when compared to cell culture without treatment (medium). Cells untreated (medium) and treated with anti-CD3 and anti-CD28 stimuli were used as negative and positive controls, respectively. (**A**) ZnO nanocrystals at a dose of 25 μg/mL reduced the proliferation of *L.b.* promastigotes from day 4, while in the treatment with ZnO:5Ag (**B**) NCPs, there was no significant difference on any of the days analyzed. (**C**) ZnO:9Ag NCPs reduced *L.b* parasite proliferation from the third day of treatment from the lowest dose tested. (**D**) Treatment with ZnO:11Ag NCPs caused a decrease in the proliferation of promastigote forms from day 4 at a dose of 25 μg/mL and on day 5 at a dose of 12.5 μg/mL.

When observing the effect of these nanoformulations against the promastigote forms in *L.b.*, it was seen that the ZnO nanocrystal and the ZnO:11Ag NCPs (25 μg/mL dose) inhibited the proliferation of the parasites from the fourth day of treatment (Figure 2A,D). In addition, ZnO:9Ag NCPs also showed leishmanicidal activity even at lower doses and with shorter exposure time to nanomaterials, inhibiting *L.b* strains from 6.25 μg/mL on the third day of treatment (Figure 2C). ZnO:5Ag NCPs did not significantly inhibit parasite proliferation (Figure 2B).

### 3.4. Ag-ZnO/AgO NCPs Reduce L. braziliensis Intracellular Amastigotes

**Figure 3:**
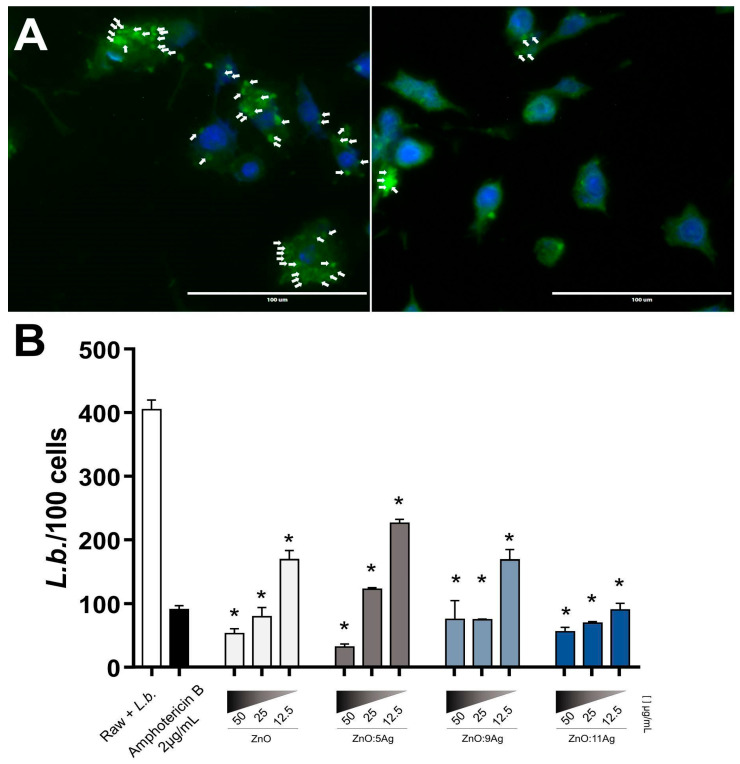
ZnO:9Ag and ZnO:11Ag NCPs reduce L. braziliensis intracellular amastigotes. (**A**) Intracellular *L.b.* amastigotes were evaluated by staining with DAPI (blue) and IgG anti-*Leishmania*+anti-IgG-FITC (green) and counting at least 200 cells/treatment. White arrows indicate intracelular amaastigores. Representative images: Left panel—Non-treated culture; Right panel—ZnO:11Ag—50 μg/mL. (**B**) RAW 264-7 were infected with *L.b.* (MOI 10:1) for 12 h and treated with different nanoformulations at doses of 50, 25 and 12.5 μg/mL for 72 h. The results are expressed as the mean ± SEM. The Mann–Whitney test was applied, and values of *p* < 0.05 (*) were considered significant when compared to non-treated cultures. Culture without treatment (medium). Cells treated with Amphotericin B (2 µg/mL) were used as treatment control.

Based on the effect of Ag-ZnO/AgO NPCs to directly kill and reduce *L.b.* promastigote proliferation along days of in vitro cultivation, we evaluated the leishmanicidal potential against intracellular amastigotes, the real parasite stage able to infect mammalian cells. All evaluated Ag-ZnO/AgO NPCs showed high leishmanicidal activity (Figure 3). Especially ZnO:9Ag and ZnO:11Ag were able to reduce cellular parasitism at lower concentrations and were previously characterized as less toxic to host cells with higher selectivity index. These results demonstrate the potential of these Ag-ZnO/AgO NPCs act in infected macrophages, the most important site of leishmania-host battlefield.

### 3.5. Ag-ZnO/AgO Activate LTCD4+ and LTCD8+ and Induce Low Cytotoxic Levels at the Lowest Concentrations

**Figure 4:**
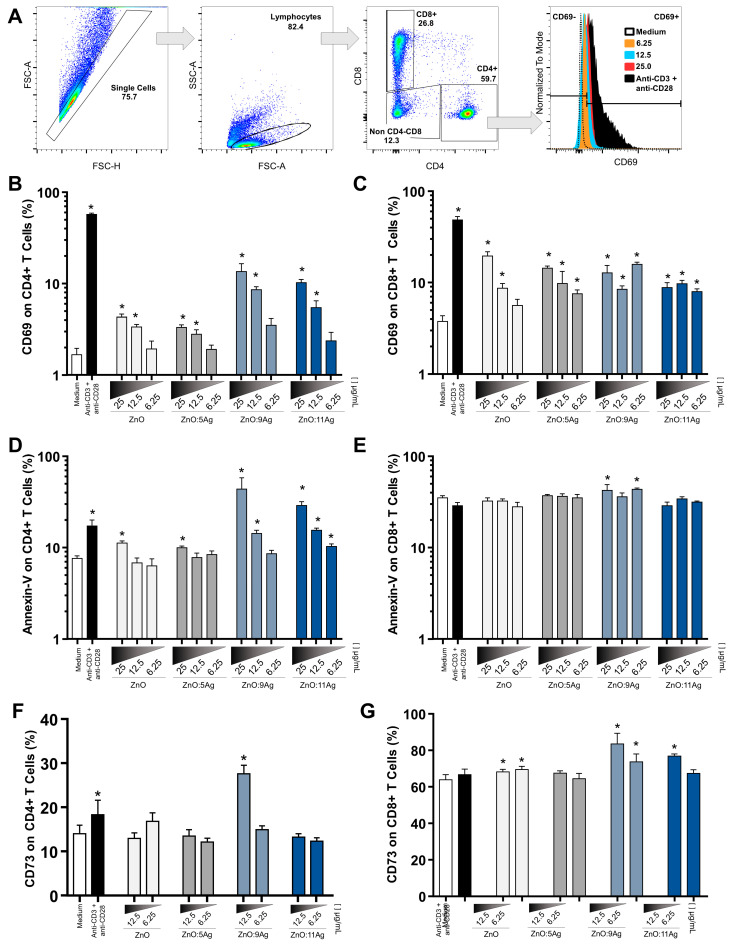
Effect of nanoformulations on the expression of CD69, annexin-V and CD73 molecules in T lymphocytes. (**A**) Representative images of gate strategy based on (left to right): removal of cell aggregate, isolation of Lymphocytes based in size and cell complexity, isolation of CD4+ and CD8+ T cells and determination of cell marker expression (%).The results are expressed as the mean fluorescence intensity ± SEM. The unpaired *T* test (CD69 and annexin) and the Mann–Whitney test (CD73) were used for comparison with untreated cells (medium), with *p* values <0.05 (*) being considered significant. Cells stimulated with anti-CD3 and anti-CD28 were used as positive controls. (**A**) Flow Cytometry analysis strategy. (**B**) From the dose of 12.5 μg/mL, all tested nanomaterials increased the expression of CD69 in CD4+ T cells, with ZnO:9Ag and ZnO:11Ag being the most efficient NCPs. (**C**) Cell activation of CD8+ CD69+ T lymphocytes was significantly higher in all treatments and doses, with the exception of treatment with ZnO nanocrystals at 6.25 μg/mL. (**D**) All nanoformulations at a dose of 25 μg/mL increased annexin-V labeling in CD4+ T cells when compared to the untreated control group. (**E**) In CD8+ cells, it was seen that only ZnO:9Ag increased annexin production. (**F**) ZnO:9Ag NCPs at a dose of 12.5 μg/mL increased the expression of CD73 in CD4+ T cells. (**G**) In CD8+ T lymphocytes, ZnO nanocrystals, ZnO:9Ag and ZnO:11Ag NCPs at a dose of 12.5 μg/mL increased the expression of this molecule.

Previously, it was observed that metallic NPs are able to interact with cells of the innate and adaptive immune systems [41,42,43,44]. Here, the aim was to explore the interaction of ZnO nanocrystals as well as Ag-ZnO/AgO NCPs with T lymphocytes, as they are fundamental to modulate macrophage function and control of infectious agents. First, the activation of LTCD4+ and LTCD8+ was evaluated based on the expression of CD69 by Flow Cytometry (Gate strategy represented in Figure 4A) in cultures of treated PBMCs, and all nanomaterials were able to increase this marker of cell activation (Figure 4B,C).

Furthermore, to assess the cytotoxicity of the treatment on CD4+ and CD8+ T cells, annexin-V staining (Figure 4D,E) was performed on the PBMC cultures to indicate signs of cell damage. The results suggest that toxicity was higher in CD4+ T lymphocytes treated with ZnO nanocrystals and ZnO:5Ag NCPs at a dose of 25 μg/mL, with ZnO:9Ag NCPs at a concentration of 12.5 μg/mL and with ZnO:11Ag NCPs at the lowest dose (Figure 4D). In CD8+ T cells, only ZnO:9Ag NCPs increased the expression of this molecule when compared to the control group (Figure 4E). Although the dose of 25 μg/mL stimulated the expression of CD69, the annexin-V labeling in lymphocytes was high, evidencing probable cell damage in healthy cells, and for this reason, this dose was excluded from the other analyses.

After excluding the treatment of nanomaterials at a dose of 25 μg/mL, the CD73 molecule was used to evaluate another indication of cell expression. The results showed that CD4+ T cells treated with ZnO:9Ag NCPs at a dose of 12.5 μg/mL (Figure 4F) exhibited increased expression of CD73. Furthermore, CD8+ T lymphocytes treated with ZnO nanocrystals, ZnO:9Ag and ZnO:11Ag NCPs (even at the lowest doses tested) were able to increase this protein, indicative of cell activation (Figure 4G).

Together, these data suggest that, at adequate doses, the study Ag-ZnO/AgO may have immunomodulatory activities and acceptable cellular toxicity to T cells.

### 3.6. Expression of TNFR1 and TNFR2

Establishing an effective inflammatory response is essential for controlling infectious agents. For this, it is necessary to have, in addition to soluble factors, receptors to stimulate these immune cell responses, as is the case with TNFR1 and TNFR2 receptors.

**Figure 5:**
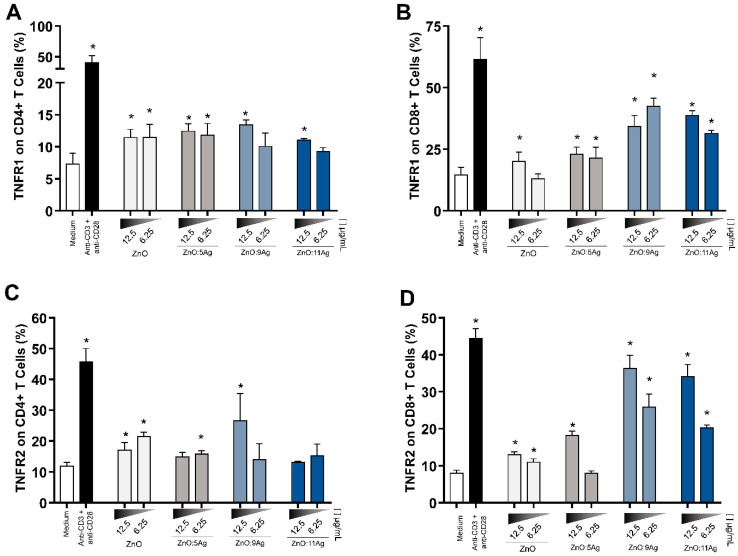
Nanomaterials promote increased expression of TNF-α receptors in T lymphocytes. Expression levels of TNFR1 (Figure 4A,B) and TNFR2 (Figure 4C,D) were determined by flow cytometry in CD4+ and CD8+ T lymphocytes from treated PBMCs for 72 h. The results are expressed as the mean fluorescence intensity ± SEM. The Mann–Whitney test was applied, and values of *p* < 0.05 (*) were considered significant when compared to untreated cell culture (medium). Untreated cells (medium) and cells treated with anti-CD3 and anti-CD28 stimuli were used as negative and positive controls, respectively. (**A**) All treatments and doses (except ZnO:11Ag NCPs at 6.25 μg/mL) upregulated TNFR1 expression in CD4+ T lymphocytes. (**B**) In CD8+ T cells, all nanoformulations (except ZnO nanocrystals at 6.25 μg/mL) upregulated the expression of TNFR1, with this production being more significant in cells treated with ZnO:9Ag NCPs. (**C**) CD4+ TNFR2+ T lymphocytes were increased in cultures treated with ZnO nanocrystals (at 12.5 μg/mL and 6.25 μg/mL), ZnO:5Ag NCPs (at 6.25 μg/mL) and ZnO:9Ag NCPs (at 12.5 µg/mL). (**D**) CD8+ T cells expressed more TNFR2 than the control group in all tested nanoformulations in a dose-dependent manner, with the ZnO:9Ag and ZnO:11Ag NCPs at 12.5 μg/mL having the highest activity.

Therefore, when evaluating these markers in CD4+ T cells, we observed that TNFR1 was upregulated using the highest dose of nanomaterials, with ZnO nanocrystals and ZnO:5Ag NCPs also expressed at the lowest dose (6.25 μg/mL) (Figure 5A). Furthermore, TNFR2 was more highly expressed in these cells when treated with ZnO nanocrystals at both doses, while for ZnO:5Ag and ZnO:9Ag NCPs, this increase was observed only at the highest and lowest doses, respectively (Figure 5C).

All nanoformulations and doses tested increased the expression of TNFR1 and TNFR2 in CD8+ T lymphocytes (except for ZnO nanocrystals at a dose of 6.25 μg/mL and for TNFR1 and ZnO:5Ag NCPs at a dose of 6.25 μg/mL for TNFR2) (Figure 5B,D), and treatments with ZnO:9Ag and ZnO:11Ag NCPs were the most potent in increasing the expression of these receptors.

### 3.7. NPCs Differentially Regulate Immunoregulatory Molecules in CD4+ and CD8+ T Cells

**Figure 6:**
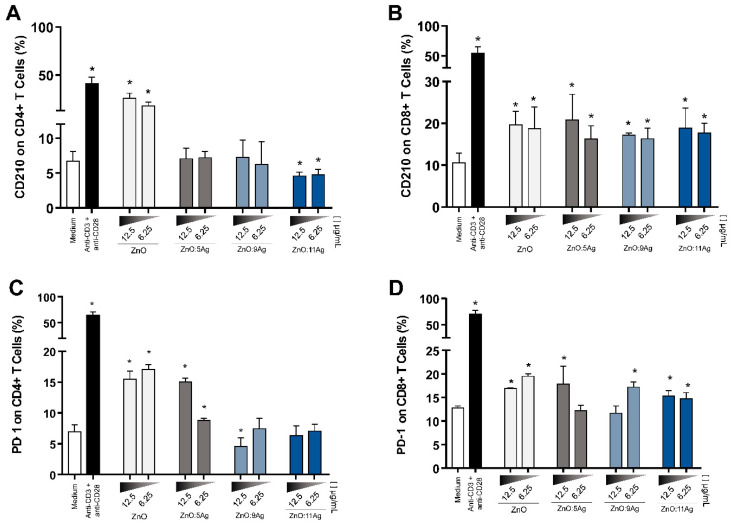
NPCs differentially impact the expression of CD210 and PD-1 in T lymphocytes. Expression of CD210 (Figure 5A,B) and PD-1 (Figure 5C,D) was performed by flow cytometry on CD4+ and CD8+ T lymphocytes from PBMCs treated for 72 h, and these results are expressed as the mean fluorescence intensity ± SEM. The Mann–Whitney test was applied, and values of *p* < 0.05 (*) were considered significant when compared to cell culture without treatment (medium). Cells without treatment (medium) and cells treated with anti-CD3 and anti-CD28 stimuli were used as negative and positive controls, respectively. (**A**) ZnO nanocrystals increased CD4+ 210+ T cells in cultures when ZnO:11Ag NCPs were added. (**B**) In CD8+ T lymphocytes, all treatments upregulated the expression of CD210. (**C**) CD4+ T cells treated with ZnO nanocrystals and ZnO:5Ag NCPs increased the expression of PD1, while ZnO:9Ag NCPs downregulated this expression. (**D**) All tested nanoformulations increased PD-1 expression in CD8+ T lymphocytes but in a dose-dependent manner.

To assess the immunoregulatory potential, T cells from treated PBMCs were evaluated for CD210 and PD-1 expression. The results showed that ZnO nanocrystals increased, in CD4+ T cells, the expression of IL-10 receptors at the two doses tested, while ZnO:5Ag and ZnO:9Ag had no significant difference in the production of these receptors. ZnO:11Ag NCPs decreased CD4+ CD210+ T lymphocytes when compared to the control group (Figure 6A). In contrast, all nanoformulations increased CD210 expression on CD8+ T cells (Figure 6B), which suggests that these cells are prone to advance to a regulatory profile when compared to CD4+ T cells.

Furthermore, evaluating the expression of PD-1 in these T cells, it was observed that ZnO and ZnO:5Ag NCPs increased this expression, while ZnO:9Ag NCPs at the highest dose were the only NCPs capable of negatively regulating the expression of this molecule (Figure 6C). In CD8+ T lymphocytes, nanomaterials increased PD-1 expression, but not in a dose-dependent manner (Figure 6D).

### 3.8. NCPs Induce a Proinflammatory Cytokine Secretion Profile and Increase Nitric Oxide (NO)

**Figure 7:**
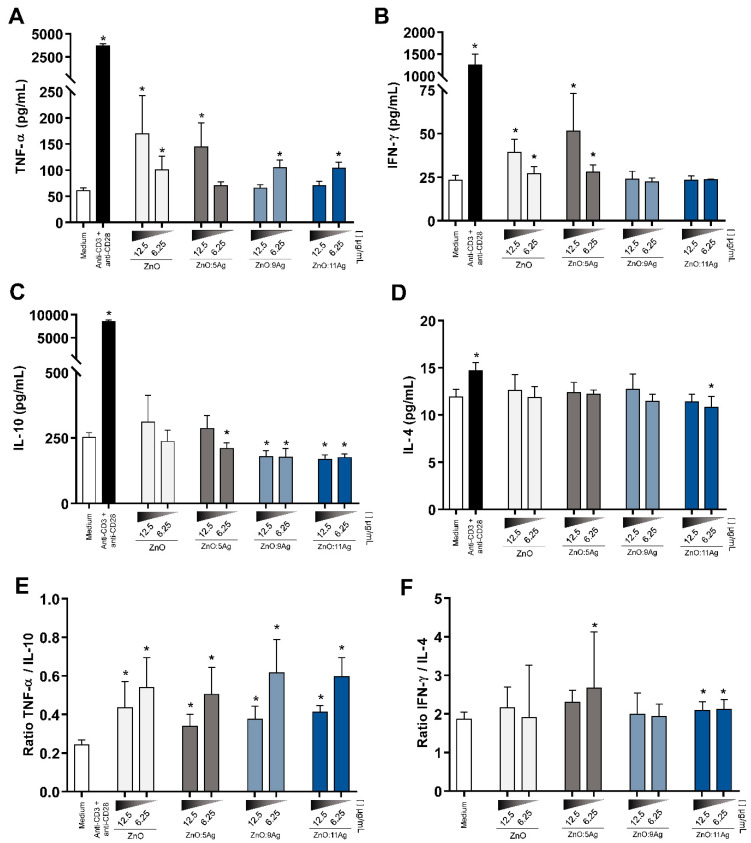
Cytokine production by treated PBMCs. The levels of TNF-α, IL-10, IFN-γ and IL-4 were evaluated by ELISA in the supernatant of cultures of PBMCs treated with different nanoformulations at doses of 12.5 μg/mL and 6.25 μg/mL for 72 h. The results are expressed as the mean ± SEM in picograms per milliliter (pg/mL). The Mann–Whitney test was applied, and values of *p* < 0.05 (*) were considered significant when compared to cell culture without treatment (medium). Cells without treatment (medium) and cells treated with anti-CD3 and anti-CD28 stimuli were used as negative and positive controls, respectively. (**A**) TNF-α levels were increased when treated with ZnO nanocrystals, ZnO:9Ag and ZnO:11Ag NCPs at the two doses tested, as well as treatment with ZnO:5Ag at 12.5 μg/mL. (**B**) The level of IFN-γ was upregulated in ZnO nanocrystals and ZnO:9Ag NCPs at both doses. (**C**) IL-10 quantification was lower in the treatment with ZnO:5Ag NCPs at 6.25 μg/mL and in both doses in the treatment with ZnO:9Ag and ZnO:11Ag NCPs. (**D**) For IL-4, only ZnO:11Ag NCPs at 6.25 μg/mL decreased the production of this cytokine. (**E**) All nanomaterials had increased levels of TNF-α compared to IL-10 levels. (**F**) ZnO:5Ag at 6.25 μg/mL and ZnO:11Ag (two doses tested) positively regulated IFN-γ levels when compared with IL-4 levels.

When analyzing the cytokines present in the supernatant of treated PBMCs, the levels of TNF-α (Figure 7A) in the cultures treated with ZnO nanocrystals and ZnO:5Ag NCPs were dose-dependently increased, whereas the increase in the dose in the treatment of ZnO:9Ag and ZnO:11Ag NCPs decreased the production of this cytokine. IFN-γ levels (Figure 7E) increased in cultures of PBMCs treated with ZnO nanocrystals and ZnO:5Ag NCPs at the two doses tested and in a dose-dependent manner. Furthermore, IL-10 in these cultures was downregulated when treated with ZnO:5Ag NCPs at the lowest dose and in ZnO:9Ag and ZnO:11Ag NCPs at both doses. IL-4 levels were upregulated in ZnO nanocrystals and ZnO:5Ag NCPs at some tested doses.

Based on these results, the relationship between the quantification of TNF-α and IL-10 levels (Figure 7C) and between IFN-γ and IL-4 levels (Figure 7F) was determined. In the evaluation of TNF-α/IL-10, there was a predominance of TNF-α in relation to IL-10 in all treatments when compared to the control group without treatment. In the relationship between IFN-γ/IL-4, there was a predominance of IFN-γ over IL-4 in ZnO:5Ag NCPs at a dose of 6.25 μg/mL and in ZnO:11Ag NCPs at doses of 12.5 μg/mL and 6.25 μg/mL.

**Figure 8:**
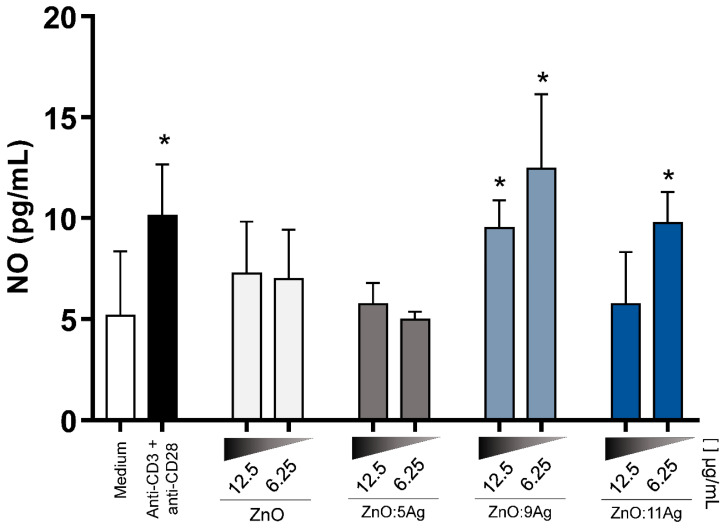
ZnO:9Ag and ZnO:11Ag NCPs increased NO production. NO production was evaluated by the modified Griess method in the supernatant of PBMC cultures treated with different nanoformulations at doses of 12.5 μg/mL and 6.25 μg/mL for 72 h. The results are expressed as the mean ± SEM in picograms per milliliter (pg/mL). The Mann–Whitney test was applied, and values of *p* < 0.05 (*) were considered significant when compared to cell culture without treatment (medium). Cells without treatment (medium) and cells treated with anti-CD3 and anti-CD28 stimuli were used as negative and positive controls, respectively. NO production was increased in cultured cells treated with ZnO:9Ag and ZnO:11Ag NCPs, especially at the lowest dose.

Additionally, considering the importance of nitric oxide (NO) in combating intracellular pathogens, its production in these supernatants was quantified. After treatment, it was found that the ZnO:9Ag and ZnO:11Ag NCPs increased the production of this metabolite, with the lowest dose, 6.25 μg/mL, the best NO inducer (Figure 8).

## 4. Discussion

In the last decade, nanomaterials composed of metal and metallic oxides have been the target of studies that have shown them promising in combating pathogens [45]. These can be used pure, doped with ions or in conjunction with other nanocrystals forming NCPS to improve the established activities and raise the possibility of other biological activities [46]. In this context, the use of nanoformulations of containing ZnO, Ag-doped ZnO and AgO nanocrystals as already been studied due to their biological properties that give them several important activities, including microbicidal action [31,47,48], and immunomodulatory activity [49].

Knowing that the ZnO nanoformulation has important biological actions, our group set out to understand how the different forms of this NP would behave in toxicity assays. When comparing amorphous ZnO NPs and ZnO nanocrystals in healthy cells, it was shown that the nanocrystals were less toxic [50]. Seeking to potentiate the effect of these ZnO nanocrystals, doping with Ag ions was performed, forming Ag-doped ZnO NPs. Furthermore, we sought to further improve the NPs by adding AgO in different amounts to form Ag-ZnO/AgO NCPs. As different forms of synthesis and processing of nanomaterials—even if they come from the same common material—can produce nanoformulations with different properties [36], we emphasize that the synthesis and dispersion methods used here were different from those already described in the literature, making these nanomaterials unpublished.

Furthermore, TL is an important public health problem that requires new and better forms of prophylaxis and treatment, and we hypothesized that the nanomaterials synthesized and characterized here could be effective in combating this set of diseases. In fact, our findings showed that Ag-ZnO/AgO NCPs exert action against *L.b* in promastigote forms and showed good selectivity for parasites when compared with toxicity in murine macrophages. We observed that the amount of AgO added to NCPs influences this activity. It was noticed that Ag-ZnO/AgO NCPs with 49% AgO (ZnO:5Ag NCPs) do not present toxic action against *L.b* strains, but when we increase the proportion of AgO to 65% (ZnO:9Ag NCPs), the leishmanicidal activity reaches its peak maximum. When the proportion of AgO is increased to 68% (ZnO:11Ag NCPs), the action against *L.b* drops, indicating that there is an ideal ratio of Ag-ZnO and AgO NP in NCPs to obtain better leishmanicidal action.

Even with the notorious leishmanicidal activity exerted by the ZnO:9Ag NCPs, they were also the most toxic to both healthy murine macrophages and T lymphocytes from the PBMC culture. Despite the high rate of annexin-V expression by the treated lymphocytes, the selectivity index showed satisfactory values. As in *L.b*. infection, macrophages are the main cells involved [51] and that toxicity for this cell type was satisfactory for ZnO:9Ag NCPs, we can assume that the best route of administration of this nanomaterial is topical to avoid systemic toxicity. We emphasize that future experiments in in vivo assays using these NCPs in *L.b* infection will be essential to collect more data on this.

In addition, we proposed to establish immunomodulatory activity in cell cultures from healthy donors. These immune cells were treated with the nanoformulations, and we sought to extrapolate the results to an *L.b*. infection. In analysis, the importance of T cells in the fight against TL is well described [5], and the activation of T cells for this process is fundamental. CD69 is one of the first surface proteins expressed during lymphocyte activation and behaves as a costimulatory molecule of the activation and proliferation processes of this cell type [46]. Additionally, the CD73 ectoenzyme is also involved in these processes and provides proliferation signals and influences the migration of activated T cells [52]. The nanomaterials tested increased the expression of these proteins, suggesting that they are able to activate cellular immune mechanisms to establish a robust cellular immune response that may be beneficial in *L.b* [53]. In the future, it is important to understand which pathways are involved in these processes to strengthen this correlation.

The organism’s homeostasis process relies on a delicate balance between pro- and anti-inflammatory mediators, a dichotomy widely studied in leishmaniasis in terms of resistance and host susceptibility to infection [54]. Soluble inflammatory mediators such as TNF-α, IFN-γ and NO, as well as their receptors, are essential to stimulate *L.b* clearance [50,51], and parasite persistence in lesions correlates with immune failure [55]. In this context, we observed that PBMCs treated with ZnO:9Ag and ZnO:11Ag NCPs are more likely to go toward the inflammatory response profile when compared to the activity of ZnO nanocrystals and ZnO:5Ag NCPs. ZnO:9Ag and ZnO:11Ag NCPs increased TNFR1 and TNFR2 expression and TNF-α and NO levels and decreased IL-10 levels in the treated cultures. Interestingly, treatment with ZnO nanocrystals and ZnO:5Ag NCPs did not increase the production of these variables, but they were the only nanocomposites capable of increasing IFN-γ levels in PBMC cultures. Recently, [56] showed that Ag-ZnO/AgO NCPs with 56% AgO presented anti-inflammatory and healing action on mouse wounds, stimulating increased collagen synthesis at the injury site and other healing processes. This shows that Ag-ZnO/AgO NCPs exert different mechanisms of immune system modulation and that the amount of AgO in these NCPs has a profound impact on their biological action.

Furthermore, we believe it is important to evaluate how these nanomaterials influence the expression of PD-1 in T cells, since this protein is associated with the exhaustion of immune cells and evasion of immune mechanisms, in addition to the fact that its increase is related to LT progression [57]. By showing that NCPs, especially ZnO:9Ag NCPs, decrease the expression of this molecule, we suggest that this may favor immune mechanisms to combat *L.b* infection, since, as shown by [58], genes associated with exhaustion cellular—including those related to PD-1—were upregulated in biopsies from patients infected with *L.b* and, also, circulating CD4+ and CD8+ T cells with increased expression of PD-1.

We understand the need to balance this inflammatory response, considering that the uncontrolled increase in inflammatory mediators is correlated with the severity of TL and tissue damage [59,60,61]. The results obtained by [45] exemplify this issue well by showing that CD8+ T cells are predominant in biopsies of patients with skin ulcers. These data raise the hypothesis that even though these cells are in an inflammatory profile, probably associated with tissue damage by the release of granzymes and perforins, they are not the most suitable to fight the parasites that cause TL. Our results provide relevant data on this issue because, even though several inflammatory mechanisms are elevated in the cultures of treated cells, CD8+ T cells show increased levels of inhibitory markers, such as PD-1 and CD210. With this, we suggest that, in the context of *L.b* infection, CD8+ T cells may present less production of inflammatory mediators that could cause excessive damage to healthy tissues.

## 5. Conclusions

This study reports the synthesis of Ag-doped ZnO nanocomposites with AgO nanocrystals, which for the first time were subjected to evaluation in leishmanicidal and immunomodulatory activities. We suggest that Ag-ZnO/AgO NCPs—in particular, ZnO:9Ag NCPs and ZnO:11Ag NCPs have therapeutic potential to treat infections caused by *L.b*. since they promote a leishmanicidal response with a satisfactory selectivity index, reduced proliferation of promastigotes and amastigotes, in addition to an immune response focused on the inflammatory profile with activation of LTCD4+ and LTCD8+, demonstrating the ability to activate T cells at adequate doses. It showed an increase in inflammatory cytokines such as IFN-γ and TNF-α as well as the production of nitric oxide. All doses tested increased the expression of TNFR1 and TNFR2, suggesting activation of efficient inflammatory responses in killing *L.b*. We emphasize that future in vivo and in vitro studies will be important to understand the other mechanisms involved in treatments with these NCPs. However, our study demonstrates an innovative leishmanicidal strategy in the treatment of leishmaniasis.

## Figures and Tables

**Table 1:** Selectivity index of treatments on RAW 264.7 and *L.b*.

Nanomaterial	Target Cell	CC50/IC50 (μg/mL)	Selectivity Index
ZnO nanocrystal	RAW 264.7	1232	1.33
	*L.b*	927.9	
ZnO:5Ag NCPs	RAW 264.7	1330	3.35
	*L.b*	397	
ZnO:9Ag NCPs	RAW 264.7	412.6	52.03 *
	*L.b*	7.93	
ZnO:11Ag NCPs	RAW 264.7	312.4	20.38 *
	*L.b*	15.33	

The determination of IC50 and CC50 was performed using the rezazurin assay after 24 h of treatment, and subsequently, the selectivity index was calculated (IC50/CC50), and values greater than 10 were considered relevant (*).

## Data Availability

The datasets generated during and/or analysed during the current study are available from the corresponding author on reasonable request.
